# Comparison of Machine Learning Models for Predicting Recurrent Lumbar Disc Herniation After Percutaneous Endoscopic Lumbar Discectomy

**DOI:** 10.3390/jcm15145728

**Published:** 2026-07-22

**Authors:** Yang Tian, Bin Zhang, Jiao Li, Xiangyang Guo, Junsheng Duan, Kai Wang, Shuiqing Li

**Affiliations:** 1 Department of Anesthesiology, Peking University Third Hospital, Beijing 100191, China; 2 Department of Anesthesiology, Tibet Autonomous Region People’s Hospital, Lhasa 850002, China; 3 Department of Pain Medicine, Peking University Third Hospital, Beijing 100191, China

**Keywords:** machine learning, recurrent lumbar disc herniation, percutaneous endoscopic lumbar discectomy, predictive model, risk factors

## Abstract

**Background**: Recurrent lumbar disc herniation (rLDH) significantly impairs outcomes following percutaneous endoscopic lumbar discectomy (PELD). Accurate individualized risk prediction remains challenging. This study aimed to develop and compare multiple machine learning models for predicting rLDH within two years post-surgery. **Methods**: A retrospective cohort of 1483 patients undergoing single-level PELD was analyzed. The primary outcome was symptomatic, magnetic resonance imaging-confirmed rLDH requiring reintervention. Candidate predictors included demographic, surgical, and radiographic parameters. The dataset was stratified by outcome and randomly split into training (70%, *n* = 1038) and validation (30%, *n* = 445) sets. Feature selection utilized univariate screening (*p* < 0.2) and least absolute shrinkage and selection operator regression. Six machine learning algorithms were trained and optimized via grid search. Performance was evaluated using the area under the receiver operating characteristic curve (AUC), F1-score, Brier score, calibration and decision curve analysis (DCA). Model interpretability was assessed using SHapley Additive exPlanations (SHAP). **Results**: The overall recurrence rate was 4.25% (63/1483). Logistic regression achieved the optimal F1-score (0.286), while light Gradient Boosting Machine (LightGBM) demonstrated superior discrimination (AUC = 0.768). DCA indicated clinical utility primarily at low threshold probabilities (<10%). SHAP analysis identified increased sagittal range of motion as the strongest risk factor, followed by reduced facet orientation, advanced age, type II Modic changes, and Michigan State University zone C. **Conclusions**: This study presents an exploratory, internally validated machine learning framework for rLDH risk stratification. While LightGBM demonstrated moderate discriminative ability, model sensitivity was constrained by the inherent rarity of recurrence events, precluding its use as a definitive standalone screening tool. Notably, clinical utility was restricted to low threshold probabilities (<10%), supporting a focused role in identifying high-risk subgroups for intensified preoperative counseling and postoperative monitoring. Beyond elucidating key radiological and demographic risk factors, our findings underscore that rigorous external prospective validation and probability calibration are indispensable before any future clinical deployment.

## 1. Introduction

Percutaneous endoscopic lumbar discectomy (PELD) has revolutionized the surgical management of lumbar disc herniation (LDH), offering a minimally invasive alternative to conventional open procedures. Its benefits, including reduced paraspinal muscle injury, shorter hospital stays, and faster return to daily activities, are well-documented [1,2]. However, recurrent lumbar disc herniation (rLDH) at the index level remains a formidable and relatively common complication, with reported incidence rates ranging from 3% to 15% across different studies and follow-up durations [3,4,5]. Our medical center cohort exhibits a recurrence burden consistent with this established epidemiological range (detailed quantitative findings are presented in the Results section), confirming that rLDH represents a tangible and frequent clinical challenge rather than a rare anomaly. This recurrence not only negates the initial benefits of surgery but also subjects patients to renewed pain, disability, the psychological burden of a failed procedure, and the risks and costs associated with revision surgery [6].

The quest to identify patients at high risk for rLDH has been a focus of spine research. Numerous studies have employed traditional statistical methods, primarily logistic regression, to pinpoint individual risk factors. These have been broadly categorized into: (1) patient-specific factors such as advanced age, higher body mass index (BMI), and smoking history [7,8]; (2) disc morphology and degeneration characteristics, including the Pfirrmann grade of disc degeneration and the presence and type of Modic changes in the vertebral endplates [9,10,11]; and (3) biomechanical and anatomical parameters like lumbar lordosis (LL) and segmental mobility [12,13]. Notably, the Michigan State University (MSU) classification system, which provides a standardized morphological description of herniation location and severity, has shown promise in correlating with surgical outcomes, though its link to recurrence is still being elucidated [14,15].

Despite these efforts, clinical prediction remains challenging. Traditional multivariate models often assume linear relationships and may struggle with complex interactions between a high number of potential predictors. Furthermore, the typically low incidence of rLDH poses a significant statistical challenge, often leading to models with high specificity but poor sensitivity—missing the very high-risk patients they aim to identify [16]. Machine learning, a subset of artificial intelligence, offers a paradigm shift. Machine learning algorithms are theoretically capable of handling high-dimensional data and capturing non-linear relationships without strict a priori assumptions regarding linearity or multicollinearity [17]. In spine surgery, machine learning has been successfully applied to predict outcomes such as surgical site infection, length of stay, and patient-reported outcomes [18,19]. A few pioneering studies have begun applying machine learning to predict complications or reoperation after lumbar surgery [20,21]. However, a comprehensive, head-to-head comparison of multiple machine learning algorithms specifically for predicting rLDH after PELD is lacking. Determining the optimal algorithm and understanding the dominant predictive features within a machine learning framework is crucial for developing clinically useful tools. While machine learning improves predictive performance, its “black-box” nature hinders clinical translation. Therefore, we integrated Shapley Additive exPlanations (SHAP) analysis to deconstruct the model logic. This approach quantifies each predictor’s contribution, clarifying how specific radiological and demographic features drive recurrence risk, thereby translating algorithmic outputs into interpretable risk insights to support clinician–patient counseling.

Therefore, this study aimed to: (1) develop and internally evaluate six distinct machine learning models for predicting rLDH within two years after primary single-level PELD; (2) identify and compare the optimal model based on comprehensive performance metrics; and (3) perform an in-depth analysis of feature importance to elucidate the key clinical and radiological determinants of recurrence, thereby translating model output into actionable clinical insights.

## 2. Materials and Methods

### 2.1. Study Design and Population

This retrospective cohort study analyzed consecutive patients undergoing single-level PELD at a tertiary medical center. The period from August 2021 to December 2022 denotes the dates of the index surgery. Inclusion criteria comprised: (1) confirmed diagnosis of LDH; (2) failure of strict conservative management exceeding 3 months; and (3) single-level PELD procedure. Exclusion criteria included: (1) incomplete clinical data; (2) concurrent lumbar spinal stenosis; (3) coexisting spondylolisthesis; and (4) history of previous lumbar surgery.

To ensure methodological rigor, the study protocol was prospectively registered in the Chinese Clinical Trial Registry on 17 December 2024. Crucially, all study procedures—including data extraction and outcome ascertainment—were initiated strictly after this registration date. Patients were subsequently followed for a minimum of two years postoperatively, with the final follow-up visits occurring in December 2024. The primary outcome was defined as symptomatic, MRI-confirmed rLDH requiring reintervention, characterized by: (1) symptomatic recurrence within 2 years after surgery; (2) MRI-confirmed disc herniation at the previously operated segment; and (3) subsequent surgical intervention (revision PELD or open discectomy). The study was conducted in accordance with the Declaration of Helsinki and approved by the Institutional Review Board. The requirement for informed consent was formally waived due to the retrospective design.

### 2.2. Data Collection and Variable Definitions

Data were extracted from electronic medical records and the hospital’s picture archiving and communication system. The general characteristics encompassed clinical parameters (sex, age, BMI, smoking history, American Society of Anesthesiologists [ASA] physical status classification, anesthesia method, surgical approach, and operative time), disc herniation characteristics (Michigan State University [MSU] zone, MSU grade, surgical segment, and migrated herniation), and radiologic parameters including disc height index (DHI), LL, sacral slope (SS), endplate curvature angle (ECA), facet orientation (FO) and facet tropism (FT), sagittal range of motion (sROM), lumbar facet joint osteoarthritis (LFOA), Pfirrmann grade of disc degeneration, and Modic changes.

The MSU classification system utilizes two metrics: the MSU zone (locations A, B, C, and AB) and the MSU grade (Grades 1–3) [14]. According to sagittal MRI criteria, migrated disc herniations were classified into four zones (Zone 1–4) based on the craniocaudal direction and migration distance relative to the disc space [22]. The DHI sROM, FO, and FT were derived from Shi’s protocol [11]. The sROM was calculated as the angular difference (°) between flexion and extension at the surgical level, measured via superior/inferior endplate tangents. On axial computed tomography (CT) or MRI, lines tangent to bilateral superior articular processes form angles (α, β) with the midsagittal plane, where FO = (α + β)/2 and FT = |α − β|/2. ECA was measured on lateral radiographs by connecting the summit and endpoints of the lumbar vertebral endplate arc [23]. LFOA severity was graded 0–IV using CT/MRI criteria established by Weishaupt et al., evaluating facet joint degeneration [24]. Surgical-level disc degeneration grade was assessed via Pfirrmann grading on T2-weighted sagittal MRI [25]. Modic changes were classified as type I (low T1 and high T2), type II (high T1 and high T2), and type III (low T1 and T2) [11]. All radiographic measurements were performed by two independent, blinded surgeons using standard protocols, with disagreements resolved by consensus. To evaluate inter-observer reliability, a random subset of 40 patients (20 from the training cohort and 20 from the validation cohort) was independently re-assessed by the same two surgeons who performed the original measurements. All re-assessments were conducted under identical imaging protocols, with both surgeons blinded to patient outcomes and to each other’s results. Intraclass correlation coefficients (ICCs) were calculated using a two-way random-effects model with absolute agreement for single measures (ICC (2,1)) for continuous variables, while Cohen’s kappa (κ) was used for categorical variables. ICC (2,1) coefficients ≥ 0.75 and κ values ≥ 0.60 were considered indicative of good measurement reliability. Reliability assessment was performed prior to model development and did not influence variable selection.

### 2.3. Data Preprocessing and Feature Selection

The dataset was randomly split into a training set (70%) and a validation set (30%) using the train–test split function from scikit-learn, with stratification based on the outcome variable to preserve the same recurrence ratio in both sets.

A two-stage variable selection strategy was employed to balance dimensionality reduction with the retention of potentially relevant predictors. Initially, univariate comparisons were performed between recurrence and non-recurrence groups. Given the low event rate, a liberal threshold (*p* < 0.2) was adopted to preserve variables with modest univariate signals that might contribute meaningfully in multivariable or non-linear modeling frameworks [26,27,28]. Variables meeting this criterion were then entered into a least absolute shrinkage and selection operator (LASSO) logistic regression with 10-fold cross-validation to identify the final predictor set.

To prevent data leakage, all data preprocessing and feature scaling were conducted strictly within the training set. Specifically, the standardization parameters (mean and standard deviation) were estimated exclusively from the training data and subsequently applied to transform both the training and validation sets. No information from the validation set was used during the preprocessing phase.

### 2.4. Machine Learning Model Development and Evaluation

Six machine learning algorithms were implemented. These included: (1) logistic regression with L2 regularization and class weighting; (2) Random Forest with balanced class weighting; (3) Support Vector Machine (SVM) with an RBF kernel; (4) eXtreme Gradient Boosting (XGBoost), which employed scaled positive weighting and regularization terms; (5) light Gradient Boosting Machine (LightGBM) with balanced class weighting; and (6) Gradient Boosting via the Hist Gradient Boosting Classifier with class-weight support. Systematic hyperparameter tuning was conducted for each algorithm using 5-fold cross-validation with F1 optimization.

Model performance was assessed using area under the receiver operating characteristic curve (AUC), recall, and F1-score. Post hoc threshold optimization was performed by scanning probability thresholds from 0.1 to 0.9 in 0.01 increments on the validation set to maximize the F1-score. Calibration curves were plotted to evaluate the consistency between predicted probabilities and actual outcomes, with the Brier score serving as a quantitative indicator where lower values represent better calibration. Clinical utility was quantitatively evaluated using decision curve analysis (DCA) to estimate the net benefit across a range of clinically reasonable probability thresholds.

To enhance the clinical interpretability of the model, multiple analytical methods were employed for in-depth feature importance assessment. For tree-based models (e.g., Random Forest, Gradient Boosting, XGBoost, and LightGBM), SHAP analysis was utilized to quantify each feature’s contribution to individual predictions based on game theory, producing visualizations including SHAP summary plots and feature importance rankings by mean absolute SHAP values. For logistic regression, standardized coefficient magnitudes were used as direct indicators of feature importance, visualized via coefficient bar plots. For non-linear SVM models, permutation importance was applied by evaluating performance degradation after randomly shuffling feature values.

### 2.5. Statistical Analysis

Categorical variables were compared using the Chi-squared test, while continuous variables were compared using Student’s *t*-test or the Mann–Whitney U test based on their distribution (normality assessed by D’Agostino’s K^2^ test). All statistical analyses were conducted in Python 3.12, primarily utilizing open-source libraries such as scikit-learn, XGBoost, LightGBM, SHAP, matplotlib, and seaborn. A random seed of 42 was set to ensure reproducibility of the results. A significance level of α = 0.05 was set for all tests, and all *p*-values were two-sided.

## 3. Results

### 3.1. Patient Characteristics and Cohort Description

**Figure 1:**
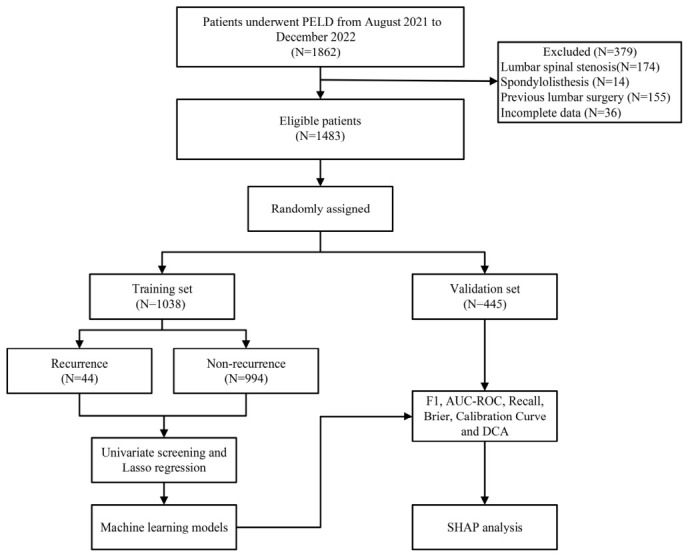
Flowchart of the study population. PELD, percutaneous endoscopic lumbar discectomy; Lasso, least absolute shrinkage and selection operator; AUC, area under curve; DCA, decision curve analysis; SHAP, SHapley Additive exPlanations.

The final cohort comprised 1483 patients. The overall incidence of symptomatic rLDH within 2 years was 4.25% (63/1483). After stratified splitting, the training set contained 1038 patients (recurrence: 44, 4.2%) and the validation set contained 445 patients (recurrence: 19, 4.3%) (Figure 1). Patient characteristics in the training and validation sets were shown in Table 1 and Table 2.

### 3.2. Feature Selection and Machine Learning Model Performance

**Figure 2:**
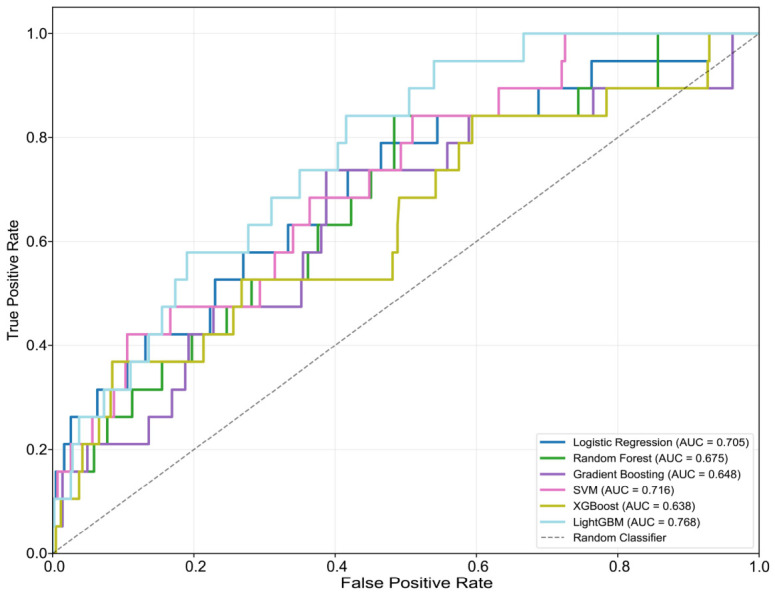
ROC curves. ROC, receiver operating characteristic; AUC, area under curve; SVM, Support Vector Machine; XGBoost, eXtreme Gradient Boosting; LightGBM, light Gradient Boosting Machine.

Univariate analysis in the training set identified five variables with *p* < 0.2: MSU zone, Modic changes, age, FO, and sROM. LASSO regression was performed on the above five variables for feature selection. Ultimately, seven features were retained, including three continuous variables (age, FO, and sROM) and four binary-encoded features derived from categorical variables. Specifically, the categorical variables MSU zone and Modic changes were one-hot encoded, yielding the following features: MSU zone A, MSU zone C, no Modic changes, and Type II Modic changes. These features will be used for subsequent predictive model construction. The performance of all six models on the validation set using their respective optimized thresholds is summarized in Figure 2 and Table 3. Of the models evaluated, LightGBM achieved the highest AUC, indicating the strongest discriminative ability, while logistic regression yielded the highest F1-score and optimal balance between precision and recall. Calibration curves for LightGBM within the predicted probability range of 0 to 0.4 showed good alignment with the ideal line at low probabilities, but the curve remained generally below the reference line across most intervals, suggesting a slight underestimation of absolute risk. LightGBM yielded a Brier score of 0.050, indicating acceptable overall calibration. Calibration assessment of the LightGBM model yielded a slope of 1.62 and an intercept of −3.24. For comparison, the logistic regression model yielded a slope of 1.74 and an intercept of −3.87. DCA across the threshold range of 0 to 0.3 demonstrated that LightGBM provided positive net benefit only within the narrow range of 0 to 0.1, whereas net benefit became negative at higher thresholds and was inferior to the treat-none strategy, underscoring that clinical utility is strictly restricted to very low probability thresholds (see Supplementary Figures S1 and S2).

### 3.3. Feature Importance and SHAP Interpretability Analysis

**Figure 3:**
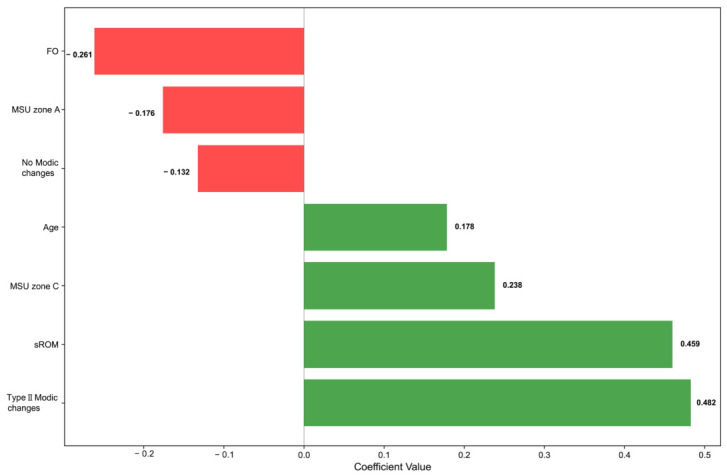
Logistic regression feature coefficients. FO, facet orientation; MSU, Michigan State University; sROM, sagittal range of motion.

Logistic regression analysis identified Type II Modic changes (coefficient: +0.482) and sROM (coefficient: +0.459) as the most significant positive predictors, while FO was identified as the most significant negative predictor (coefficient: −0.261). In addition, MSU zone C (coefficient: +0.238) and age (coefficient: +0.178) also emerged as statistically meaningful positive predictors (Figure 3).

**Figure 4:**
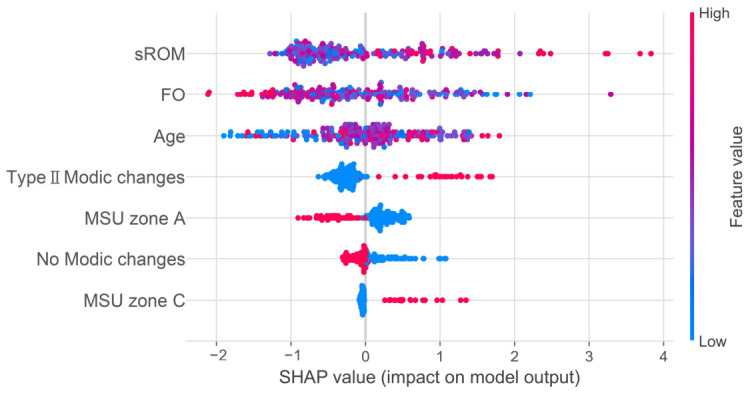
LightGBM SHAP summary plot. LightGBM, light Gradient Boosting Machine; SHAP, SHapley Additive exPlanations; sROM, sagittal range of motion; FO, facet orientation; MSU, Michigan State University.

Figure 4 presented the aggregated SHAP summary plot for the LightGBM model, illustrating feature contributions to the predicted recurrence probability. Red and blue points indicated high and low feature values, respectively, with point density reflecting sample concentration. Key findings revealed that sROM exhibited the widest SHAP value range (approximately −1.5 to 3.5), where high values were strongly associated with increased risk. In contrast, high FO values corresponded to reduced risk (SHAP < 0), while low FO values increased it. For age, MSU zone C, and Type II Modic changes, high values consistently elevated risk (SHAP > 0), whereas low values were linked to lower risk.

## 4. Discussion

In the present study, we developed and compared multiple machine learning models to predict rLDH following PELD. A clear trade-off emerged between model interpretability and discriminative performance. Logistic regression, with an optimal threshold of 0.78 dictated by the low event prevalence, offered the highest transparency. Its coefficients directly quantified risk associations; for example, Type II Modic changes (+0.482) and segmental range of motion (+0.459) increased risk, whereas facet orientation (−0.261) reduced it. However, its discriminative performance was modest (AUC = 0.705), reflecting limitations in modeling complex, non-linear interactions and highlighting the need for cautious interpretation.

By contrast, LightGBM achieved the best discrimination (AUC = 0.768) with an optimal threshold of 0.31, better capturing non-linear relationships in this clinical setting. Nevertheless, DCA showed that LightGBM yielded a positive net benefit only within a narrow threshold range (0–0.1); beyond this range, the net benefit turned negative and remained inferior to the “treat-none” strategy. This restricted utility window indicates that these models should not be used to guide treatment decisions at standard or higher probability thresholds. Notably, the overall modest F1 scores and limited recall reflect the inherent difficulty of predicting rare events (~4.25% incidence) and caution against overstating the model’s predictive utility. Consequently, these models should be interpreted strictly as clinical risk stratification aids to identify high-risk subgroups for intensified preoperative counseling or modified surgical techniques, rather than as definitive criteria for determining surgical eligibility. With a recall of only 0.263, LightGBM would fail to flag approximately three out of four recurrent cases, posing an unacceptable risk of false reassurance. Therefore, any attempt to use these models to exclude patients from surgery or guide prophylactic interventions would be premature and potentially unsafe. Instead, they are best positioned to facilitate enhanced patient counseling regarding radiological risk profiles and to prioritize higher-risk subgroups for selective postoperative monitoring. SHAP explanations further provide a nuanced understanding of recurrence risk, offering a basis for individualized counseling and selective postoperative monitoring.

Ranking highly in the LightGBM model, an increased sROM is a potent and biomechanically plausible risk factor for rLDH after PELD. This finding is strongly supported by the existing literature. A retrospective matched case–control study by Shi et al. specifically identified a large sROM as a significant independent radiological risk factor for rLDH after percutaneous transforaminal endoscopic discectomy [11]. Similarly, Kong et al., in their analysis of recurrent L4/5 disc herniation, reported a large sROM as an independent significant risk factor in their multivariate Cox regression model [12]. The biomechanical rationale is that a hypermobile segment subjects the surgically created annular defect to greater repetitive shear and tensile forces during daily activities, thereby potentially impeding healing and facilitating re-herniation of residual nucleus pulposus. This concept aligns with the instability hypothesis of disc failure. Therefore, the assessment of sROM via preoperative dynamic flexion–extension radiographs is not merely a research parameter but represents a valuable yet potentially underutilized component of routine clinical risk stratification.

A critical observation from our comparative analysis is the apparent rank discrepancy of key predictors—particularly sROM and FO—between the standardized coefficients of logistic regression and the SHAP values derived from LightGBM. While logistic regression identified Type II Modic changes and sROM as the foremost positive predictors based on main effects, the LightGBM-SHAP analysis revealed a more nuanced hierarchy shaped by non-linear interactions. The SHAP dependence plot for sROM interacted with FO (Supplementary Figure S3), which elucidates this discrepancy: the contribution of sROM to predicted risk is not constant but is profoundly modulated by FO. High sROM values conferred substantial risk (positive SHAP values) primarily when FO was high (red dots); conversely, this risk-enhancing effect diminished or reversed when FO was low (blue dots). This interaction is inherently captured by SHAP but remains invisible to Logistic Regression unless explicitly encoded as interaction terms—which would necessitate prior clinical hypotheses.

This divergence aligns with established explainable AI literature demonstrating that SHAP values and regression coefficients serve distinct explanatory purposes [29,30]. Logistic regression coefficients quantify isolated main effects under assumptions of linearity and feature independence. In contrast, SHAP values attribute contributions conditional on feature interactions and non-linear dependencies. In tree ensembles like LightGBM, sROM’s ascendance to the top SHAP rank reflects its pivotal role in higher-order interactions—particularly with FO—rather than a deficiency in the linear model. Such non-linear anatomical interactions are biologically plausible, suggesting that the prognostic value of sROM is contingent upon the underlying spinal morphology (FO).

Modic changes, especially type II, were consistent with a growing body of literature [10,31]. Modic changes represent a state of chronic, reactive vertebral endplate and bone marrow pathology; this state is closely linked to disc degeneration and a pro-inflammatory microenvironment [32]. This environment may impair the healing capacity of the annulus fibrosus post-discectomy. Furthermore, type II changes might indicate a more advanced, irreversible degenerative state that fundamentally compromises the disc-vertebra functional unit’s integrity, predisposing to recurrence. The precise etiology and pathophysiological mechanisms in relation to rLDH remain incompletely elucidated and warrant further investigation.

Advanced age was a well-established and significant risk factor for rLDH after PELD. A meta-analysis demonstrated that patients experiencing recurrence were significantly older, with a weighted mean difference of 9.95 years compared to non-recurrent patients [7]. Supporting this, a prognostic model study further identified an age threshold, reporting that patients aged 45 years or older had a more than twofold increased risk of recurrence (OR = 2.17) [8]. The underlying pathophysiology is multifactorial. Age-related declines in disc proteoglycan and water content reduce tissue turgor and resilience, while degenerative annular delamination and fissures create pathways for re-herniation of residual disc material. Furthermore, the systemic diminution of tissue repair and healing capacity in older individuals may impede proper closure of the surgical annular defect, leaving a persistent structural weakness that predisposes to recurrence [7,8].

The variable FO was selected by LASSO and was an important feature in LightGBM and logistic regression. The association between a lower FO and an increased risk of rLDH was supported by multiple lines of evidence. A clinical study confirmed FO as an independent risk factor, with risk rising as FO decreases [33]. Biomechanical analysis further clarified that, especially when combined with FT, a lower FO concentrates stress on the ipsilateral disc, explaining the heightened re-herniation risk [34]. Additionally, machine learning models have identified lower FO as a key predictive feature for rLDH after endoscopic surgery, affirming its role in a multifactorial etiology [35]. Collectively, evidence from the present study and prior research, spanning multiple perspectives, demonstrates that a lower, more sagittal FO is not only a clinical risk factor for rLDH but also possesses a plausible biomechanical explanatory mechanism. This underscores its potential value as a preoperative imaging biomarker for risk stratification.

Previous studies reported mixed or non-significant associations between herniation location and recurrence [4,5]. In contrast, our integrated analysis, which combines LightGBM and logistic regression models, underscores that MSU zone C is a consistent risk factor, while zone A may have a more context-dependent role. This finding is consistent with the relatively high recurrence rate observed for zone C in our training set (six of 67 cases), which likely results from a combination of anatomical complexity, technical challenges, and biomechanical vulnerability [36]. Specifically, the proximity to critical structures such as the exiting nerve root and dorsal root ganglion makes visualization difficult, while the restricted mobility of endoscopic instruments increases the risk of incomplete decompression or retained fragments.

This study has limitations. Its retrospective, single-center design may introduce selection bias and limit generalizability; external validation in diverse populations is therefore essential. The most significant limitation is the low number of recurrence events (*n* = 63), which inherently constrains model sensitivity and increases the variance of performance estimates. We are particularly concerned that only 19 recurrent cases were available within the validation set (4.3% of *n* = 445). This extreme sparsity limits the reliability of our performance metrics; statistical fluctuations inherent to small event counts increase the variance of sensitivity and calibration estimates. Consistent with this, the 95% confidence interval for LightGBM’s AUC was relatively wide (0.674–0.855), reflecting the imprecision of discriminative performance estimates derived from such a small event count. For instance, the calibration slope of 1.62 and intercept of −3.24 in LightGBM, while informative, may be unstable due to the limited number of observed events. Moreover, although SHAP analysis offers mechanistic insights, the attribution of feature importance in such a data-sparse environment should be interpreted cautiously, as it may be sensitive to the specific composition of this small validation cohort. The modest recall across all algorithms reflects this fundamental constraint and precludes the use of these models as definitive screening tools. Notably, the negative net benefit observed in decision curve analysis above a 10% threshold underscores that clinical implementation at higher probabilities would result in net harm.

A pertinent illustration of this sampling variance is provided by operative time. While univariate screening in the training set did not identify a significant association (*p* = 0.629), the same variable reached statistical significance in the validation set (*p* = 0.008) and was consequently omitted due to our two-stage feature selection protocol. Although a post hoc full-variable LASSO confirmed that operative time contributed negligibly compared to dominant predictors (e.g., sROM, Modic changes), this discrepancy underscores the risk of discarding variables with context-dependent relevance via univariate pre-filtering in low-event cohorts. Additionally, the model currently uses preoperatively available data; incorporating intraoperative findings (e.g., annular tear size, completeness of decompression) and postoperative factors (e.g., early return to heavy labor) could enhance predictive power. Furthermore, while we addressed imbalance via threshold optimization, techniques such as SMOTE or ensemble methods designed for imbalance could be explored in future iterations.

A critical methodological limitation pertains to threshold selection. Optimal thresholds were identified post hoc on the same hold-out validation set used for final performance reporting. This approach risks optimistic bias, potentially inflating F1-scores and precision/recall estimates—an effect amplified by the small number of events, where minor reclassifications of even 1–2 cases can substantially shift optimal thresholds. Given the limited number of events and the inherent instability of model calibration noted above, prospective recalibration in a fully independent external dataset is indispensable prior to any clinical application. Future validation studies should aim for at least 100 recurrent events to ensure stable estimation of discrimination, calibration, and clinical utility metrics, as recommended for prediction model validation [37].

Beyond statistical performance, the practical utility of a predictive model lies in its judicious integration into clinical workflows—primarily as a supplementary aid for risk communication rather than an autonomous decision-making tool. To facilitate the translation of our findings, we provide open access to a user-friendly prediction tool based on the final LightGBM model. This tool allows clinicians to input key radiological parameters via a standardized template and obtain individualized risk estimates without requiring computational expertise. The deployment package, including source code and execution scripts, is freely available as Supplementary Material at https://doi.org/10.17605/OSF.IO/Y3XCF.

## 5. Conclusions

This study presents an exploratory, internally validated machine learning framework for rLDH risk stratification. While LightGBM demonstrated moderate discriminative ability, model sensitivity was constrained by the inherent rarity of recurrence events, precluding its use as a definitive standalone screening tool. Notably, clinical utility was restricted to low-threshold probabilities (<10%), supporting a focused role in identifying high-risk subgroups for intensified preoperative counseling and postoperative monitoring. Beyond elucidating key radiological and demographic risk factors, our findings underscore that rigorous external prospective validation and probability calibration are indispensable before any future clinical deployment.

## Figures and Tables

**Table 1:** Comparison of clinical characteristics in the training and validation sets.

Variables	Training Set		*p*	Validation Set		*p*
	Non-Recurrence (*n* = 994)	Recurrence (*n* = 44)		Non-Recurrence (*n* = 426)	Recurrence (*n* = 19)	
Age (years)	41 (32, 54)	46 (37, 58)	0.037	43 (33, 54)	43 (34, 49)	0.993
BMI (kg·m^−2^)	23.7 (21.7, 25.6)	24.0 (22.2, 27.2)	0.204	23.6 (21.6, 25.6)	23.5 (22.1, 24.9)	0.970
Sex, n (%)			0.712			0.506
Male Female	593 (59.7) 401 (40.3)	28 (63.6) 16 (36.4)		247 (58.0) 179 (42.0)	13 (68.4) 6 (31.6)	
Smoking, n (%)	50 (5.0)	1 (2.3)	0.637	14 (3.3)	3 (15.8)	0.030
Anesthesia method, n (%)			0.261			1.000
Local anesthesia General anesthesia	558 (56.1) 436 (43.9)	29 (65.9) 15 (34.1)		236 (55.4) 190 (44.6)	11 (57.9) 8 (42.1)	
Surgical approach, n (%)			0.255			0.937
PEID PETD	483 (48.6) 511 (51.4)	17 (38.6) 27 (61.4)		195 (45.8) 231 (54.2)	8 (42.1) 11 (57.9)	
Surgical segment, n (%)			0.441			0.181
L3/4 L4/5 L5/S1	96 (9.7) 451 (45.4) 447 (45.0)	2 (4.5) 23 (52.3) 19 (43.2)		50 (11.7) 179 (42.0) 197 (46.2)	1 (5.3) 12 (63.2) 6 (31.6)	
Operative time (minutes)	81 (61, 103)	78 (57, 99)	0.629	84 (66, 106)	65 (49, 79)	0.008
ASA classification, n (%)			0.858			0.930
1 2 3	581 (58.5) 402 (40.4) 11 (1.1)	23 (52.3) 20 (45.4) 1 (2.3)		235 (55.2) 185 (43.4) 6 (1.4)	11 (57.9) 8 (42.1) 0 (0.0)	

Data are presented as n (%) or median (Q1, Q3). BMI, body mass index; ASA, American Society of Anesthesiologists; PEID, percutaneous endoscopic interlaminar discectomy; PETD, percutaneous endoscopic transforaminal discectomy.

**Table 2:** Comparison of radiological characteristics in the training and validation sets.

Variables	Training Set		*p*	Validation Set		*p*
	Non-Recurrence (*n* = 994)	Recurrence (*n* = 44)		Non-Recurrence (*n* = 426)	Recurrence (*n* = 19)	
MSU zone, n (%)			0.182			0.785
A B AB C	363 (36.5) 372 (37.4) 198 (19.9) 61 (6.1)	12 (27.3) 18 (40.9) 8 (18.2) 6 (13.6)		136 (31.9) 164 (38.5) 90 (21.1) 36 (8.5)	4 (21.1) 8 (42.1) 5 (26.3) 2 (10.5)	
MSU grade, n (%)			0.310			0.023
1 2 3	120 (12.1) 462 (46.5) 412 (41.4)	8 (18.2) 22 (50.0) 14 (31.8)		56 (13.1) 194 (45.5) 176 (41.3)	6 (31.6) 10 (52.6) 3 (15.8)	
Migrated herniation, n (%)	107 (10.8)	6 (13.6)	0.725	55 (12.9)	7 (36.8)	0.009
DHI	0.3 (0.3, 0.4)	0.3 (0.3, 0.4)	0.746	0.3 ± 0.1	0.3 ± 0.1	0.903
LL (°)	37.3 ± 10.6	36.2 ± 11.7	0.562	37.9 ± 10.4	37.6 ± 8.3	0.900
SS (°)	31.8 (27.8, 35.7)	31.8 (24.9, 35.8)	0.481	31.5 (27.6, 35.2)	28.6 (25.7, 32.1)	0.018
Superior ECA (°)	162.4 ± 6.8	163.1 ± 6.4	0.509	162.4 ± 6.8	161.5 ± 5.6	0.502
Inferior ECA (°)	173.1 ± 5.2	172.4 ± 5.4	0.488	173.4 ± 5.1	173.3 ± 3.6	0.848
FO (°)	48.7 ± 8.4	46.3 ± 7.8	0.048	49.3 ± 7.5	53.0 ± 8.3	0.069
FT (°)	5.4 (4.0, 7.0)	5.5 (3.3, 6.7)	0.970	5.4 (4.0, 6.7)	6.5 (3.5, 9.4)	0.176
sROM (°)	8.1 ± 2.0	9.3 ± 2.9	0.007	8.0 ± 1.9	9.9 ± 2.7	0.006
Pfirrmann grade ≥ 4, n (%)	697 (70.1)	31 (70.5)	1.000	313 (73.5)	13 (68.4)	0.824
LFOA, n (%)	915 (92.1)	42 (95.5)	0.592	394 (92.5)	16 (84.2)	0.381
Modic changes type, n (%)			<0.001			0.014
None I II III	678 (68.2) 130 (13.1) 163 (16.4) 23 (2.3)	18 (40.9) 5 (11.4) 21 (47.7) 0 (0.0)		293 (68.8) 53 (12.4) 63 (14.8) 17 (4.0)	8 (42.1) 2 (10.5) 8 (42.1) 1 (5.3)	

Data are presented as n (%) or means ± standard deviation, or median (Q1, Q3). MSU, Michigan State University; DHI, disc height index; LL, lumbar lordosis; SS, sacral slope; ECA, endplate concave angle; FO, facet orientation; FT, facet tropism; sROM, sagittal range of motion; LFOA, lumbar facet joint osteoarthritis.

**Table 3:** Performance of machine learning models on the validation set.

Model	Best Threshold	AUC (95% CI)	Brier (95% CI)	Recall (95% CI)	F1 (95% CI)
Logistic Regression	0.78	0.705 (0.578–0.827)	0.193 (0.177–0.208)	0.263 (0.105–0.474)	0.286 (0.103–0.474)
Random Forest	0.65	0.675 (0.543–0.792)	0.147 (0.138–0.157)	0.158 (0.000–0.316)	0.240 (0.000–0.462)
Gradient Boosting	0.77	0.648 (0.510–0.777)	0.062 (0.049–0.075)	0.158 (0.000–0.316)	0.214 (0.000–0.414)
SVM	0.15	0.716 (0.600–0.826)	0.039 (0.037–0.041)	0.159 (0.000–0.368)	0.240 (0.000–0.462)
XGBoost	0.51	0.638 (0.496–0.776)	0.131 (0.123–0.140)	0.368 (0.158–0.579)	0.226 (0.100–0.358)
LightGBM	0.31	0.768 (0.674–0.855)	0.050 (0.040–0.060)	0.263 (0.105–0.474)	0.250 (0.087–0.421)

AUC, area under curve; SVM, Support Vector Machine; XGBoost, eXtreme Gradient Boosting; LightGBM, Light Gradient Boosting Machine.

## Data Availability

The original contributions presented in this study are included in the article/Supplementary Material. Further inquiries can be directed to the corresponding author (Kai Wang, talent16wang@163.com). The standalone deployment tool and associated model files are permanently archived and publicly accessible via the OSF under the following DOI: https://doi.org/10.17605/OSF.IO/Y3XCF.

## Supplementary Materials

The following supporting information can be downloaded at https://www.mdpi.com/article/10.3390/jcm15145728/s1, Supplementary Figure S1. Calibration curve of the LightGBM model. The x-axis represents predicted probability (0–0.4), and the y-axis denotes observed event probability. The diagonal line stands for perfect calibration. The LightGBM curve closely matches the ideal reference line at low predicted probabilities but lies slightly below the reference line over most probability intervals, indicating mild underestimation of absolute risk by the model. Supplementary Figure S2. DCA of the LightGBM model. The x-axis represents threshold probability ranging from 0 to 0.3, and the y-axis indicates net clinical benefit. The red dashed line and green dashed line represent the two default clinical strategies: “Treat All” and “Treat None”, respectively. The LightGBM model yielded positive net benefit merely in the narrow threshold range of 0–0.1; at higher threshold probabilities, its net benefit turned negative and was worse than the treat-none strategy. Supplementary Figure S3. SHAP dependence plot for sROM interacted with FO. Points represent patients, colored by FO (red = high, blue = low). The plot demonstrates that elevated sROM increases predicted risk (positive SHAP) mainly in the context of high FO (red dots). Conversely, when FO is low (blue dots), the risk enhancing effect of sROM attenuates or reverses. SHAP, SHapley Additive exPlanations; sROM, sagittal range of motion; FO, facet orientation. Risk Model Tool is available via the Open Science Framework (OSF): https://doi.org/10.17605/OSF.IO/Y3XCF.

## Abbreviations

rLDH	Recurrent Lumbar Disc Herniation
PELD	Percutaneous Endoscopic Lumbar Discectomy
BMI	Body Mass Index
ASA	American Society of Anesthesiologists
PEID	Percutaneous Endoscopic Interlaminar Discectomy
PETD	Percutaneous Endoscopic Transforaminal Discectomy
MSU	Michigan State University
DHI	Disc Height Index
LL	Lumbar Lordosis
SS	Sacral Slope
ECA	Endplate Curvature Angle
FO	Facet Orientation
FT	Facet Tropism
sROM	Sagittal Range of Motion
LFOA	Lumbar Facet Joint Osteoarthritis
SVM	Support Vector Machine
XGBoost	eXtreme Gradient Boosting
LightGBM	Light Gradient Boosting Machine
DCA	Decision Curve Analysis
SHAP	SHapley Additive exPlanations
