# Supplementary material for: Comparison of Machine Learning Models for Predicting Recurrent Lumbar Disc Herniation After Percutaneous Endoscopic Lumbar Discectomy
Source: J Clin Med. 2026 Jul 22;15(14):5728. doi: 10.3390/jcm15145728 (PMC13412743; doi:10.3390/jcm15145728)
Supplement: Supplementary file 1 [file jcm-15-05728-s001.zip › jcm-4397996-supplementary.pdf]

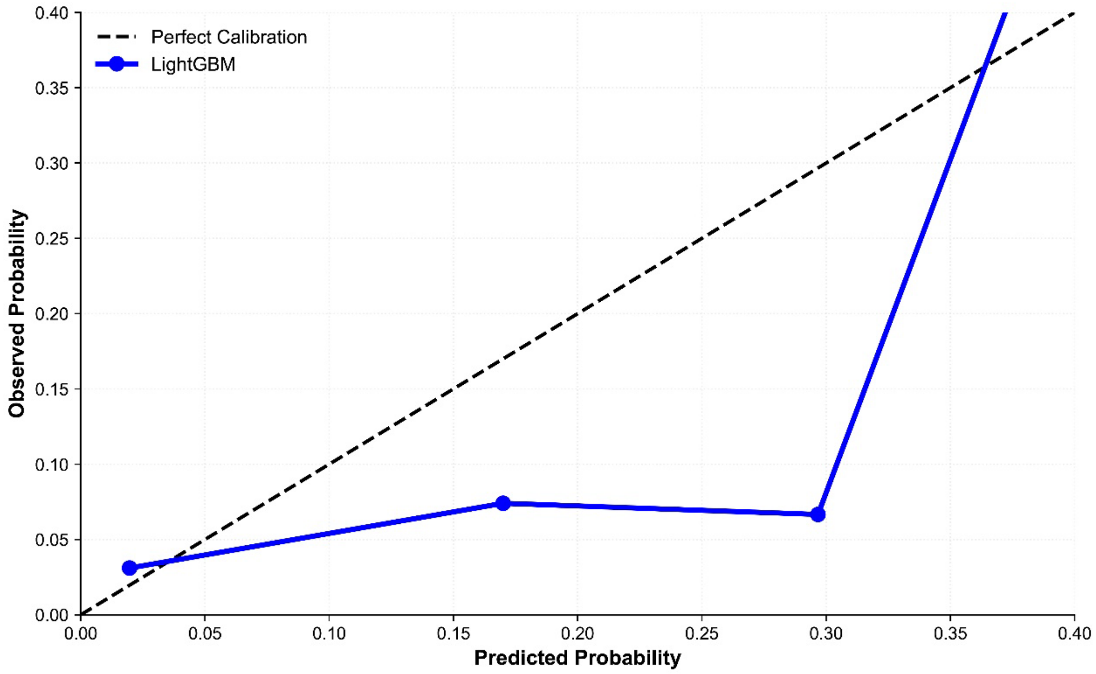

Supplementary Figure S1. Calibration curve of the LightGBM model.

The x-axis represents predicted probability (0–0.4), and the y-axis denotes observed event probability. The diagonal line stands for perfect calibration. The LightGBM curve closely matches the ideal reference line at low predicted probabilities but lies slightly below the reference line over most probability intervals, indicating mild underestimation of absolute risk by the model.

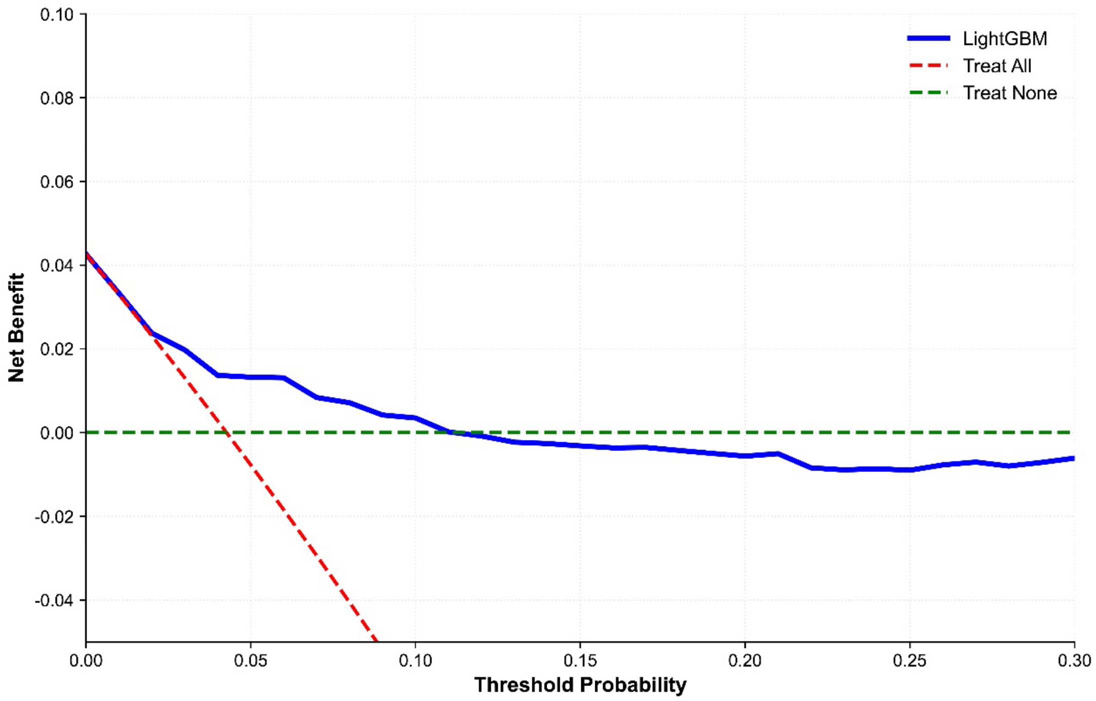

Supplementary Figure S2. DCA of the LightGBM model.

The x-axis represents threshold probability ranging from 0 to 0.3, and the y-axis indicates net clinical benefit. The red dashed line and green dashed line represent the two default clinical strategies: “Treat All” and “Treat None”, respectively. The LightGBM model yielded positive net benefit merely in the narrow threshold range of 0–0.1; at higher threshold probabilities, its net benefit turned negative and was worse than the treat-none strategy.

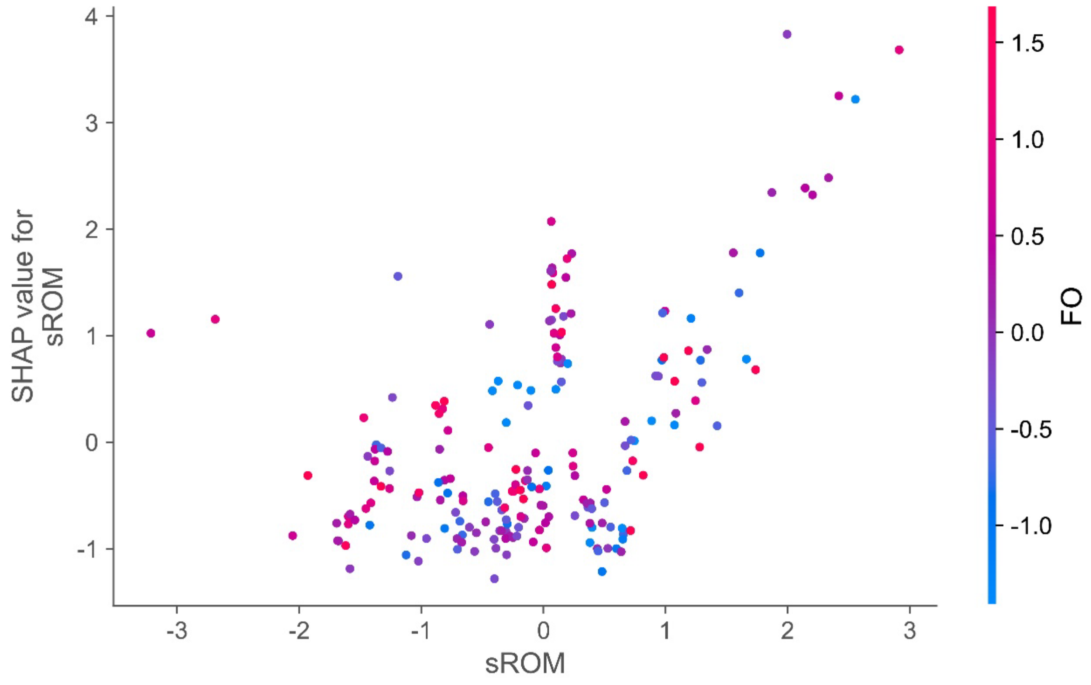

Supplementary Figure S3. SHAP dependence plot for sROM interacted with FO. Points represent patients, colored by FO (red = high, blue = low). The plot demonstrates that elevated sROM increases predicted risk (positive SHAP) mainly in the context of high FO (red dots). Conversely, when FO is low (blue dots), the risk enhancing effect of sROM attenuates or reverses. SHAP, SHapley Additive exPlanations; sROM, sagittal range of motion; FO, facet orientation.
